# Supplementary material for: A high-resolution mRNA expression time course of embryonic development in zebrafish
Source: eLife. 2017 Nov 16;6:e30860. doi: 10.7554/eLife.30860 (PMC5690287; doi:10.7554/eLife.30860)
Supplement: Supplementary file 6. [file elife-30860-supp6.zip › biolayout-clusters-files/Cluster126-genes.html]

Cluster126


# Cluster126: Genes

| Ensembl ID | Gene Name | Chr | Start | End | Biotype |
| --- | --- | --- | --- | --- | --- |
| ENSDARG00000059388 | bdh1 | 24 | 3296567 | 3304613 | protein\_coding |
| ENSDARG00000060496 | cacna1ha | 3 | 39711930 | 39812369 | protein\_coding |
| ENSDARG00000000666 | creg2 | 9 | 7249692 | 7260448 | protein\_coding |
| ENSDARG00000018716 | dgkh | 9 | 17778059 | 17952877 | protein\_coding |
| ENSDARG00000031299 | dhrs11b | 15 | 15467538 | 15472091 | protein\_coding |
| ENSDARG00000019307 | dusp5 | 22 | 29962131 | 29973677 | protein\_coding |
| ENSDARG00000059729 | si:ch211-89f7.1 | 5 | 12927609 | 12969090 | protein\_coding |
| ENSDARG00000103743 | si:dkeyp-72e1.9 | 3 | 36354768 | 36445194 | protein\_coding |
| ENSDARG00000102477 | stard4 | 21 | 223051 | 227161 | protein\_coding |
| ENSDARG00000019128 | tpm4b | 2 | 55567558 | 55584168 | protein\_coding |
